# *Helicobacter pylori* Eradication for Metachronous Gastric Cancer: An Unsuitable Methodology Impeding Broader Clinical Usage

**DOI:** 10.3389/fonc.2019.00090

**Published:** 2019-02-20

**Authors:** Alexios-Fotios A. Mentis, Efthimios Dardiotis

**Affiliations:** ^1^Public Health Laboratories, Hellenic Pasteur Institute, Athens, Greece; ^2^Department of Microbiology, University Hospital of Larissa, University of Thessaly, Larissa, Greece; ^3^Department of Neurology, University Hospital of Larissa, University of Thessaly, Larissa, Greece

**Keywords:** *Helicobacter pylori*, gastric cancer, infection, antibiotic therapy, clinical trial, pathogen

## Introduction

Recently, a study reported significant reductions in metachronous gastric cancer after *H. pylori* eradication therapy in patients with previously resected early gastric cancer. These results indicated that *H. pylori* infections benefit from treatment at any stage, thus refuting the conventional concept of the “point of no return”. Unfortunately, several methodological problems may exist in the aforementioned study that may influence the generalizability of results and conclusions and impede its broader clinical use. In this study, we discuss in detail methodological problems and rationale for caution by analyzing reported studies, aiming to help the promotion of future well-powered trials.

*Helicobacter pylori* infection is a major health concern worldwide, especially in many resource-poor countries, particularly in Africa and Latin America/Caribbean, such that more than half of the global population was infected with the pathogen *H. pylori* in 2015 (1). Gastric cancer (for which stomach adenocarcinoma accounts for around 90% of cases) is a life-threatening disease, which may be prevented by pharmacological approaches such as aspirin and non-pharmacological approaches such as gastric endoscopy (1). Basic and clinical studies have demonstrated strong associations between oncogenesis and the presence of *H. pylori* bacteria in the stomach; this includes the progression of pre-cancerous lesions (2). Remarkably, the proportion of non-cardia gastric cancer attributable to *H. pylori* increased from 74.7 to 89.0% from 2008 to 2014 (3). Furthermore, other epidemiologic factors, such as metabolic syndrome, are increasingly implicated in the etiology of gastric cancer (4). Importantly, *H. pylori* infection has also been linked to non-gastric diseases, including Parkinson's disease (5). The eradication of *H. pylori* using antibiotic therapy may prevent gastric cancer; such treatment has been implemented with varying levels of success globally (6).

## Eradication of *Helicobacter Pylori* Infection and Risk of Metachronous Gastric Cancer

The timing of interventions is often considered a key factor in determining whether cancer therapy is successful or not and whether *H. pylori* eradication is beneficial. A recent review of clinical studies revealed that *H. pylori* eradication is associated with a significantly lower risk of gastric cancer, particularly in patients with atrophic and non-atrophic gastritis, rather than in those with intestinal metaplasia; however, maximal benefit is obtained when eradication is performed during the early stages of infection (7). While this might be challenging because the infection is not typically targeted in childhood, a recent review of clinical studies confirmed that there is a general belief among healthcare practitioners that *H. pylori* eradication can prevent gastric cancer when it is administered in pre-cancerous or early cancerous stages (i.e., before a “point of no return”) (8).

In a remarkable and highly visible clinical study, Choi et al. (9) reported significant reductions in the incidence of metachronous gastric cancer after *H. pylori* eradication therapy in patients with previously resected early gastric cancer, indicating that *H. pylori* infections can benefit from treatment at any stage, thus refuting the conventional concept of the “point of no return” (1). This exciting finding seems important in convincing physicians, patients, and stakeholders, in favor of preventive *H. pylori* eradication, who might be otherwise skeptical of such measures; moreover, it generally aligns with the findings of similar recently published studies in the literature (10).

## Methodological Remarks in the Recent Clinical Trial

Previous critiques of this landmark study were focused on its scientific aspects (11). Unfortunately, we have identified several methodological problems in the study, which may impact the generalizability of the results and conclusion, regardless of whether the study is robust and/or can be replicated. Hence, there is a need for further evidence (or more rigorous clinical trials) regarding the promising role of *H. pylori* eradication in the prevention of metachronous gastric cancer.

More precisely, the study by Choi et al. (9) was a clinical trial in which ~10% of the patients developed gastric cancer, and a statistically significant difference was noted between the treated and untreated groups (*P* = 0.03). The authors reported a highly significant (*P* < 0.001) change in the atrophy grade within the corpus lesser curvature, thereby fulfilling their primary objective.

A consistent limitation of clinical studies is the inability to replicate results (frequently known as the “reproducibility crisis”); this often occurs due to low statistical power and a tendency to overinterpret statistically significant results. The researchers (9) did not report whether multiplicity corrections were used, although such statistical analyses are increasingly used in leading scientific journals (12). Combined with the reports of individual patient data, despite opposing opinions (13), we suspect that this could have helped readers to evaluate whether there is a causal association between *H. pylori* eradication and metachronous cancer reduction more accurately. A recent study demonstrated that clinical study participants are typically amenable to sharing of their individual patient data (14); the provision of such additional data would help promote detailed meta-analyses and evaluate the robustness of important results.

The corpus lesser curvature, which showed significantly less atrophy in patients who underwent *H. pylori* eradication therapy, is one of many regions where stomach adenocarcinomas exist. In the Japanese Gastric Cancer Association classification system, the corpus lesser curvature comprises three of the 12 possible lymph node stations; together with the corpus upper curvature, it is considered a part of the N1 region (15). Cancer reduction solely in the corpus lesser curvature will not necessarily result in fundamental changes with respect to TNM staging. According to Wu et al. (16), ~46% of stomach carcinomas diagnosed in the USA are located in stomach non-cardia regions; these encompass corpus lesser curvature, as well as the fundus, body, antrum, and corpus greater curvature. Non-cardia carcinoma is epidemiologically distinct from other gastric corpus cancers across different populations (17). Therefore, it is particularly notable that the authors (9) limited their analysis solely to the atrophic changes of the lesser curvature, and strong caution is advised before generalizing anti-cancer effects discerned in one form of gastric cancer from a specific population to other forms of gastric cancer and across populations. Furthermore, the authors concluded that there was a reduction in the incidence of metachronous gastric cancer and greater improvement in the grade of gastric glandular atrophy among patients who underwent *H. pylori* treatment than among patients who received placebo treatment, and this conclusion is consistent with their stated aims. However, to achieve more precise conclusions, Choi et al. (9) should have specified anatomical limitations in their conclusion, i.e., instead of the broader term “corpus,” they could have used the term “lesser curvature,” which has a stricter definition.

A particularly surprising aspect of the study by Choi et al. (9) was that only a single study pathologist performed diagnosis and biopsy evaluations, which is in direct contrast with recent trends in the cancer arena (18). To reduce potential bias in the analysis of results, especially in cases where a global conclusion is made based on clinicopathological examinations, a robust inter-rater reliability between different independent (“blinded”) pathologists should have been reported, preferably combined with parallel reporting of their level and area of expertise; moreover, Cohen's kappa coefficient might have been used as a statistic to measure inter-rater agreement (19). Further caution may be appropriate because this study concurrently considered the Vienna 4.2 [“non-invasive carcinoma (carcinoma *in situ*)”] and 4.3 (“suspicious for invasive carcinoma”) diagnostic categories. The latter evaluates the suspicion of cancer identification, and due to potential misclassification risks, they are not considered in this classification system typically (20). It is though important to acknowledge that the authors limited their primary outcome variable to gastric adenocarcinomas alone, thus facilitating comparisons with other studies. Diagnostic cultural differences are also well-known. For example, a Japanese pathologist might classify a carcinoma based on the presence of notable cytological alterations (carcinoma *in situ*), whereas an American pathologist might interpret this as high-grade dysplasia because invasions are absent.

Additionally, the authors of this study (9) should have provided a more comprehensive literature background for the 1-year clinical cutoff that they used to define metachronous cancer to provide a sense of comparability, regardless of whether all the results are recorded at 5 years; notable examples include studies by Nakajima et al. (21), Park et al. (22), Abe et al. (23), and Boda et al. (24). These contrast with the more commonly used cutoff of 6 months (Moertel definition) (25). More broadly, future clinical research would have been benefited if standardized criteria were used [akin to those in the medical terminology, as discussed by Mentis and Papavassiliou (26)]; this is particularly applicable for the design of large-scale clinical trials. Indeed, it is clearly not appropriate to compare studies with different criteria; this poses a problem when aggregating data from different studies, for example in meta-analyses. Therefore, it impacts the ability to translate research findings into clinical practice.

## Additional Patient and Pathogen Factors that Should Have Been Considered

Gastric cancer prevalence is lower in the Western hemisphere than in the Eastern hemisphere (9). An important factor associated with risk might be the *H. pylori* genotype. The CagA+, VacA s1, and VacA m1 *H. pylori* strains are associated with an increased risk of gastric cancer (27). In Asia, specific CagA polymorphisms exist; these trigger different biological mechanisms than those associated with polymorphisms found in other parts of the world (28). However, Western strains often intermix with East Asian strains; this mixing has dramatic impacts on individual disease outcome (29). Genetic screening of H. pylori would have been particularly useful in the study by Choi et al. (9) to help further identify individual patients who benefited from *H. pylori* eradication therapy, thus bridging precision medicine and public health (30). Interestingly, the association between CagA antibodies and gastric cancer development has been established for more than two decades (31). In parallel, any effects related to patient profiles, notably proinflammatory genetic makeup [reviewed in El-Omar (32)], are largely absent in the causal analysis of the clinical trial. Collectively, these data would have supported the evaluation of the relative contributions of patient and pathogen factors to the findings reported by Choi et al. (9).

## Conclusion

The hypothesis tested by Choi et al. (9) is critical for improved treatment; moreover, the importance of increasing knowledge regarding *H. pylori* eradication as preventive therapy for metachronous cancer can be cost-effective. Data have been generated for meta-analyses; however, the results cannot be generalized in their current state. Well-powered trials across different populations using the latest screening and biomarker tools available to profile individual cancer cases are needed to determine the proportion of the global population for whom *H. pylori* eradication therapy may be beneficial and cost-effective. From a clinical perspective, clinicians must also consider the risk of second primary malignancies in other body parts of patients with gastric cancer (33).

## Author Contributions

A-FM and ED conceived this opinion article. A-FM provided the initial content and draft for the manuscript, and ED enhanced the manuscript content with further literature and critical points. A-FM and ED revised the draft and approved the version to be published.

### Conflict of Interest Statement

The authors declare that the research was conducted in the absence of any commercial or financial relationships that could be construed as a potential conflict of interest.
